# Supplementary material for: Predictors of neonatal mortality in Ethiopia: Cross sectional study using 2005, 2010 and 2016 Ethiopian demographic health survey datasets
Source: PLOS Glob Public Health. 2024 Mar 18;4(3):e0002991. doi: 10.1371/journal.pgph.0002991 (PMC10947717; doi:10.1371/journal.pgph.0002991)
Supplement: Attachment — Submitted filename: Predictors of Neonatal Mortality in Ethiopia.docx [file pgph.0002991.s001.docx]

**Predictors of Neonatal Mortality in Ethiopia: Cross Sectional Study Using 2005, 2010 and 2016 Ethiopian Demographic Health Survey Demographic Health Survey Datasets**

**Review**

Overall, an important research subject, to find out what specific interventions can reduce neonatal mortality in Ethiopia.

Some constructive feedback specific to the Line#:

Line 2. Neither UNICEF nor WHO (the data cited in the paper) data support the claim that Ethiopia has the highest neonatal mortality rate in the world.

Line 3-4 is in conflict with line 11-12, depending on what is meant by “significant”

What is meant by line 14-15? Maternal ANC visits were associated with increased or decreased neonatal mortality? (I see the results in the data, but this line could be clarified)

Line 16-17 “changes in composition in the preceding birth intervals of more than 24 months” would make more sense if you write “increased birth intervals of more than 24 months…” Again in line 21, suggest adding “increased” birth intervals. Again in line 203-204, suggest remove “composition of”.

Line 157-158; would conflict have accounted for the differences in region? Was there conflict back then which prevented ANC visits? Certainly in 2020 access would have been affected by conflict.

Line 208; Do you mean “could” decrease mortality by 10%? Or “did” decrease mortality by 10%?

Line 226-228; could you briefly mention the programs and interventions implemented?

Line 228-230; I would suggest “the country's mortality rate was reduced by 11 per 1,000 live births to __________, but still there is a need to improve neonatal mortality to meet the SDG3 target of _________ (<12 per 1,000).

Line 234-239; These cited works might explain why NMR in pastoralist regions *remains bad*, but why did the NMR *get worse*? Are there fewer facilities? Has health-seeking behaviour decreased? As per Line 157-158, could it be due to conflict?

Line 271-272; I’m not sure how this logically follows. I think “low maternal and child healthcare services utilization” may have more causes than just inequality, for example, lack of facilities, barriers to access, patient factors reducing access to services (distrust of medical services, a perception that ANC visits are unimportant, limited time, etc.)

I am not a statistician, so I am taking the numbers at face value, however, there are several strange unexplained trends in the data which could be discussed:

Table 2

-the neonatal mortality rates in 2011 compared to 2005 and 2016, for example, in Tigray, Afar, and Benishangul

- Why did neonatal mortality rise over time for mothers with secondary education?

- there is no clear trend for neonatal mortality by wealth status for every level of wealth, or by maternal age, for every age tier except 20-29

Also, what is the difference between the two categories: Agrarian-Pastoralist-City and Urban-Rural? I assume Rural encompasses Agrarian and Pastoralist. If so, why combine them, because they seem to have very different outcomes – Agrarian mortality is decreasing over time, and Pastoralist is increasing, to combine them is confusing.

Table 3

- Age >35 at first birth – there are zero for 2005 and 2016, but a huge number for 2011, why? Is it because the absolute numbers are very small?

- why did NMR fall so much for females, and not for males?

Table 4

- NMR related to number of ANC visits in 2011 are the same for every number of ANC visits

- Regarding place of delivery, what is “other”. Also why is 2011 very discrepant for this criterion?

- why did NMR for c-sections increase quite a bit in 2016?

- “time postnatal check took place” shows a huge number of neonatal deaths were associates with a first postnatal check <24hr, why? Is it because most checks happen in the first 24 hrs? If you show the number of <24hr and >24hr checks as a proportion of total births it might help make sense

It would be useful if these data inconsistencies could be explained, even if your explanation is that you do not know.

Table 5 (note: it is mislabeled as Table 3 – line 196)

- what is the reason for choosing your baseline criterion in each category? For example, Agrarian, high level education, poor, young age, no antenatal visit, home birth, short birth interval, early BF, large birth weight.

- data from maternal age >35 is completely missing
