# Supplementary material for: Predictors of neonatal mortality in Ethiopia: Cross sectional study using 2005, 2010 and 2016 Ethiopian demographic health survey datasets
Source: PLOS Glob Public Health. 2024 Mar 18;4(3):e0002991. doi: 10.1371/journal.pgph.0002991 (PMC10947717; doi:10.1371/journal.pgph.0002991)
Supplement: Attachment — Submitted filename: Response to reviewers.docx [file pgph.0002991.s003.docx]

***Dear Editors***

Thank you for allowing us to submit a revision of our manuscript “Predictors of Neonatal Mortality in Ethiopia: Cross Sectional Study Using 2005, 2010 and 2016 Ethiopian Demographic Health Survey Demographic Health Survey Datasets”.

We thank the reviewers for their constructive suggestions and valuable comments. A point-by-point response to the reviewers’ comments can be found below. All page numbers apply to the “Main document clean version” document.

We look forward to hearing back from you again.

Best wishes,

Yirgalem Shibiru on behalf of the authors

Reviewers

General: Overall, an important research subject, to find out what specific interventions can reduce neonatal mortality in Ethiopia.

1. Line 2. Neither UNICEF nor WHO (the data cited in the paper) data support the claim that Ethiopia has the highest neonatal mortality rate in the world.

Reply: Thanks for your feedback. We recognized the importance of rewriting the sentence. The sentence in page one paragraph one is corrected as follows “Ethiopia is among the countries that have highest neonatal mortality in the world”(1). WHO

2. Line 3-4 is in conflict with line 11-12, depending on what is meant by “significant”

Reply: Thanks for the feedback. We revised the sentence in page one and paragraph one as follows. It now reads “Despite efforts to reduce neonatal mortality, the country has faced challenges in achieving national and global.”

3. What is meant by line 14-15? Maternal ANC visits were associated with increased or decreased neonatal mortality? (I see the results in the data, but this line could be clarified)

Reply: Thank you for highlighting the need for revising the sentence. We had modified and the sentence in page now reads as “maternal ANC visit associated with decreased neonatal morality”.

4. Line 16-17 “changes in composition in the preceding birth intervals of more than 24 months” would make more sense if you write “increased birth intervals of more than 24 months…” Again in line 21, suggest adding “increased” birth intervals. Again in line 203-204, suggest remove “composition of”.

Reply: Thank you for the suggestion. We modified the sentences in line 16- 17, line 21 and 203- 204 . It now reads “increased birth interval of more than 24 months”

5. Line 157-158; would conflict have accounted for the differences in region? Was there conflict back then which prevented ANC visits? Certainly in 2020 access would have been affected by conflict.

Reply: Thank you for your feedback. We acknowledge the potential influence of conflict on our outcome measures. However, it's important to note that we did not consider conflict as a factor in the analysis of neonatal mortality differences. This decision was based on the lack of supporting literature for this evidence and the unavailability of relevant data in our database.

6. Line 208; Do you mean “could” decrease mortality by 10%? Or “did” decrease mortality by 10%?

Reply: We appreciated your question. According to the result from the analysis "could" is the right word because it indicates the possible contribution the factor could have for the reduction of the mortality rate.

7. Line 226-228; could you briefly mention the programs and interventions implemented?

Reply: Thank you for your feedback, we recognized the importance of additional explanation about the interventions. We added the following paragraph in page 14: it now reads

“ This is despite the fact that the country has implemented community based and essential newborn care interventions under the national newborn and child survival strategy to overcome neonatal mortality for two decades. Community based newborn care aimed at actively engaging communities in promoting and preventing newborn healthcare through health extension workers ( HEWs) and health development army’s ( HDAs) at the community level. Essential newborn care services are services addressing the specific needs of newborns from birth up to 28 days and provided at health facilities level. ”

8. Line 228-230; I would suggest “the country's mortality rate was reduced by 11 per 1,000 live births to __________, but still there is a need to improve neonatal mortality to meet the SDG3 target of _________ (<12 per 1,000).

Reply: thank you for your feedback and we revised the sentence accordingly and it now reads “the country's mortality rate was reduced by 11 per 1,000 live births, from 39 per 1000 live births in 2005 to 28 per 1000 in 2016, but still there is a need to improve neonatal mortality to meet the SDG3 target of <12 per 1,000”.

9. Line 234-239; These cited works might explain why NMR in pastoralist regions remains bad, but why did the NMR get worse? Are there fewer facilities? Has health-seeking behaviour decreased? As per Line 157-158, could it be due to conflict?

Reply: Thank you for your feedback. We acknowledge the need for further explanation of increased neonatal mortality in the pastoralist region compared to other regions. As a response, we have incorporated the following details into the paragraph 3 on page 15:’’

The absence of health service delivery systems that are tailored to pastoralist lifestyles and setting may amplify the number of unmet demands and deter women in pastoralist communities from seeking healthcare during pregnancy and childbirth, and further resulting in low utilization of maternal and child healthcare , and contributing to high neonatal mortality in the regions. Furthermore, low vaccine coverage and high incidence of infectious diseases in pastoralist regions leads to neonatal mortality to get worse in the region.”

10. Line 271-272; I’m not sure how this logically follows. I think “low maternal and child healthcare services utilization” may have more causes than just inequality, for example, lack of facilities, barriers to access, patient factors reducing access to services (distrust of medical services, a perception that ANC visits are unimportant, limited time, etc.)

Reply, we appreciated your feedback and edited the paragraph on page 16_as follows:” Neonatal mortality can be preventable, but it remains a major public health problem in Ethiopia, particularly in pastoralist communities, due to low access and utilization of maternal and child health services. This emphasized the importance of improving access and barriers to maternal healthcare services to reduce neonatal mortality in the regions. Therefore, efforts are needed to prevent neonatal mortality that targets pastoralist communities.”

11. Why did neonatal mortality rise over time for mothers with secondary education?

Reply: Thank you for raising this important concern. As outlined in the results section, there is an observed increase in neonatal mortality among mothers with secondary education. However, this increase might be linked to the relatively small absolute numbers of participants who experienced our effect of interest in the study. Or Upon conducting a multivariate analysis, it was determined that maternal education status did not have a significant association with newborns deaths in our study. Consequently, it is likely that the initial observed result was influenced by confounding variables and other unexplained factors within the study.

12. there is no clear trend for neonatal mortality by wealth status for every level of wealth, or by maternal age, for every age tier except 20-29

Reply: thank you for your comment, the error was due to technical reasons and it addressed accordingly.

13. What is the difference between the two categories: Agrarian-Pastoralist-City and Urban-Rural? I assume Rural encompasses Agrarian and Pastoralist. If so, why combine them, because they seem to have very different outcomes – Agrarian mortality is decreasing over time, and Pastoralist is increasing, to combine them is confusing.

Reply: Thanks for the feedback and Agrarian-Pastoralist-City is based on the living habit of the population. Population in the agrarian regions depends on agriculture for their living, while population in the pastoralist regions are pastoralist nomad. In contrast Urban-Rural classification is based on the country requirements that define place of resident as rural and urban. Thus, it mightn’t be possible to consider agrarian and pastoralist as rural residents because some towns in the regions are considered as urban residents.

14. Age >35 at first birth – there are zero for 2005 and 2016, but a huge number for 2011, why? Is it because the absolute numbers are very small?

Reply: Thanks you for the feedback, We recognized that age > 35 at first birth are zero for 2005 and 2016 and a bi number for 2011. It is due to very small absolute number ( 0.1 for 2005, 0.2 for 2011and 0.1 2016)

15. Why did NMR fall so much for females, and not for males?

Reply: Thanks for the question. We accepted the question that crude analysis indicates the mortality fall so much for female compared to male, but multi-level regression showed that sex of the newborns did not have association with neonatal mortality. This might be due to confounders and other unexplained factor in the study.

16. NMR related to number of ANC visits in 2011 are the same for every number of ANC visits

- Regarding place of delivery, what is “other”. Also why is 2011 very discrepant for this criterion?

Reply: Thank you for your question. Other refers to place of birth that takes place neither home nor health institution, such as in the forest, car, etc, which might have been a common practice in a certain part of the country. Also, it’s a very small absolute number, 0.4%, which might be the reason for the very discrepant criterion.

17. Why did NMR for C-sections increase quite a bit in 2016?

Reply: thanks you for your question. We also recognized the increase neonatal mortality among newborns that delivered through C-section in 2016, this might be due to small absolute number, 1.5%, and crude analysis that mightn’t indicate the association between C section and newbors death. Furthermore, a previous study indicated inconsistent association of neonatal mortality and C section delivery (2)

18. “time postnatal check took place” shows a huge number of neonatal deaths were associates with a first postnatal check <24hr, why? Is it because most checks happen in the first 24 hrs? If you show the number of <24hr and >24hr checks as a proportion of total births it might help make sense

Reply, Thanks for your feedback, postnatal check within 24 hours is an absolute small number, 0.1%, which might be the reason for the high number of neonatal deaths in the crude analysis. However, multivariate analysis didn’t show the association of time postnatal care took place with newborn death, making it hard to justify the crude result. Also, since the neonatal mortality reported rate, per 1000 live births, it might be difficult to put it as a proportion.

19. What is the reason for choosing your baseline criterion in each category? For example, Agrarian, high level education, poor, young age, no antenatal visit, home birth, short birth interval, early BF, large birth weight.

Reply: Thanks for the question. References categories for the regression output are selected by default by the analysis software. Normally, the software chooses the categories with most observation as reference categories so as to have small Standard Errors (SE) of the estimated regression coefficient.

References

1.	World Health Organization. New born: improving survival and well-bieng . 2020.

2.	Yisma E, Mol BW, Lynch JW, Smithers LG. The changing temporal association between caesarean birth and neonatal death in Ethiopia: Secondary analysis of nationally representative surveys. BMJ Open. 2019;9(10):1–13.
